# The Influence of the Type of Fibers on the Reduction of the Threshold Effect in the Transition Zone of a Railway Track

**DOI:** 10.3390/ma15165730

**Published:** 2022-08-19

**Authors:** Włodzimierz Idczak, Tomasz Lewandrowski, Dominik Pokropski, Grzegorz Rogojsz, Tomasz Rudnicki

**Affiliations:** Faculty of Civil Engineering and Geodesy, Military University of Technology, 2 Gen. Sylwestra Kaliskiego Str., 00-908 Warsaw, Poland

**Keywords:** concrete additives, concrete fibers, concrete strength tests, threshold effect

## Abstract

The presented article concentrates on the influence of various concrete additives in the form of fibers on the mechanical parameters of concrete so as to obtain the effect of gradual changes in these parameters, which is very important in the transition zone of the railway track. Steel, polymer and glass fibers, as well as concrete without additives, were accepted for the study. The effect of additives on the consistency of the mixture, compressive strength, frost resistance and elastic modulus was studied. The research concerned concrete samples and models of elements of the ballastless railway surface, i.e., track slab and concrete block supports. The track slab model was made of concrete without additives, while the models of supports were made both without and with additives. The studies were carried out in laboratory conditions. As a result, the tested concrete samples with various additives were ranked so that they could be used as a material for elements of the railway surface in the transition zones of engineering facilities on railway roads, which is important from the point of view of reducing the threshold effect occurring in these zones. Detailed laboratory tests were presented, the results of these studies were discussed, and final conclusions were drawn regarding the technology of materials and the methodology of constructing the transition zones of the railway surface in order to avoid or at least reduce the threshold effect existing in such zones.

## 1. Introduction

In previous practice, concrete additives in the form of mineral ingredients or chemical admixtures were used for two reasons: (1) to obtain concretes with increased strength parameters, and (2) to use waste raw materials and to increase the recycling rate. This article describes the use of concrete additives in the form of various fibers in order to obtain a gentle change of the strength parameters of fragments of the railway surface on the section of the transition zone in order to eliminate, or at least reduce, the impact of the threshold effect on the dynamic interactions at the interface of the rail vehicle with the railway track.

### 1.1. A Brief Overview of Previous Studies

With the development of construction, there is an increase in demand for concretes with high strength parameters. Due to the occurrence of concrete shrinkage, it is not possible to increase the cement content in it indefinitely. For this reason, a number of mineral additives and chemical admixtures have been used for concrete for decades [1,2,3,4]. One of them is diffuse reinforcement in the form of fibers. Currently, such solutions are used in ceilings, tunnels, foundations, road construction and even prefabricated elements [5,6,7,8]. The main task of diffuse reinforcement is to prevent the formation of shrinkage microcracks in concrete. Moreover, fibers for concrete have a beneficial effect on mechanical and operational parameters after bonding the concrete mix. They may improve the compressive strength, as well as improve the frost resistance of concrete. Some modern techniques for concrete reinforcement with polymers were widely described in [9]. In addition, the use of additives in the form of fibers for the production of concrete has an ecological aspect, because in this case, waste materials, such as used glass or plastics, become additives. The use of used glass or plastics for the production of additives for concrete can improve the recycling rate of these materials. For example, glass recycling in Poland is about 62% and is several percent lower than the average for the European Union. In many countries, this indicator reaches up to 95%. Increasing the use of concrete additives makes it possible to increase this indicator so that as little waste as possible goes to landfills [10]. Another very interesting study, concerning the influence of certain recycled aggregates added in place of sand on the mechanical properties of concrete, is presented in [11]. Extending the life cycle of concrete used in road construction can be achieved, for example, by 100% recycling of the concrete surface even after 80 years or by using cements with a low carbon footprint. A very important element in improving the durability of the road surface is the use of fibers and chemical admixtures that increase the plasticity of the mixture and shape the structure of aeration of hardened concrete [12].

### 1.2. New Applications

The use of various concrete additives to obtain materials with different, slightly changing strength parameters has not been the subject of research described in the available literature. This type of research was initiated in the work presented in [13]. This article is a continuation of the research presented there, where a simple computational theoretical model of the interaction of a rail vehicle with a railway track, which made it possible to study dynamic phenomena arising in the zones of change of railway surface technology, was described. In the literature on the subject for calculations, a model of the railway surface is adopted, in which the rail is based on the elastic Winkler substrate characterized by the modulus of elasticity of the substrate. The validity of the adopted model was confirmed by previous research and analytical work, among others, presented in [14]. The validity of this approach has also been proven, inter alia, in [15], where a number of variants of rail, including four ways of mapping a vehicle in the form of a stream of concentrated forces, a stream of concentrated masses and streams of one- and two-mass oscillators, were analyzed. The same representations of a moving-rail vehicle were also used in [16,17,18], each time giving correct, real results. The theoretical model described in [13] was experimentally verified using laser scanning technology on active sections of the railway line. The advantages of measurements performed in this technology include high accuracy and automation, as well as the speed of measurements and the lack of the need to destroy the tested object or exclude it from operation. The main element of the measuring set included the laser scanner of the scanCONTROL LLT2610-50 series [19]. Thanks to positive experimental verification of the computational theoretical model, further theoretical analyses were possible for any/variable material technologies of surfaces in transition zones where the threshold phenomenon occurs. The threshold effect has a negative impact not only on the railway surface, but on the object that is exposed to excessive loads and vibrations as well [20,21,22]. The negative effects of the threshold effect were reduced in [13] by modifications of the railway ballast surface. In this article, concerning experimental research, an attempt is made to modify the ballastless section of the railway surface. Thanks to this, after applying the obtained set of materials in the elements of the railway surface in the transition zones: in front of and behind the railway engineering infrastructure facility, a significant reduction in the adverse effects of the threshold effect that occurs in these zones will be achieved. Thus, the next points of the article describe research on the mechanical properties of the material samples themselves, as well as tests of laboratory models of specific elements of the railway track, used in the transition zones of the railway line, made of previously tested materials.

## 2. Materials and Methods

### 2.1. Materials

The composition of the concrete mix intended for the track slab has been designed on the basis of the method of three equations in accordance with the requirements set out in the technical documents [23,24,25,26]. In the further part of the article, cement concrete intended for the track plate is marked with the symbol Z1.

Due to the fact that block supports are prefabricated elements and the design of the concrete mix is a trade secret, the components of concrete intended for block supports were selected on the basis of information obtained from the manufacturer of the ÖBB-PORR system and analogies to the requirements set out in “the Technical Conditions for the Execution and Acceptance of Prestressed Concrete Sleepers and Turnouts Id-101” [27]. In order to analyze the strength parameters in concrete intended for block supports, an additive in the form of fibers was used. Concretes in the further part of the article will be marked as:

Z2—concrete with the addition of steel fibers, Z3—concrete with the addition of polymer fibers, Z4—concrete with the addition of glass fibers, Z5—reference concrete, without the addition of fibers.

For concrete mixes, fine aggregate 0/2 from the Żabiny mine (Morąg, Poland) and granite coarse aggregate with fractions 2/8 and 8/16 from the Graniczna mine (Strzegom, Poland) were used. The concrete mix intended for the Z1 track slab was made using CEM I 42.5 N/NA cement from the WARTA company (Trębaczew, Poland), while CEM 42.5 R cement from the CEMEX company (Warsaw, Poland) was used to make the concrete mix intended for Z2–Z5 block supports. The properties of the cements used are shown in Table 1. Design assumptions for concrete mixtures are listed in Table 2 and laboratory prescriptions in Table 3 and Table 4.

The dosage of fibers in individual mixtures meets the requirements specified by the manufacturer and the standard provisions [28,29].

**Figure 1:**
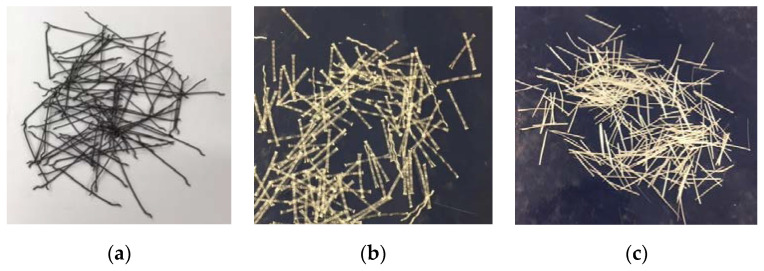
Fibers used: (**a**) steel, (**b**) polymer, (**c**) glass.

In the concrete mix intended for block supports Z2, steel fibers for concrete were used, from the Siatpol company (Majdan Stary, Poland) with a length of 50 mm in the amount of 25 kg/m^3^ of concrete mix. The concrete mix intended for Z3 block supports uses Polyex Duro polymer fibers from the Astra company (Straszyn, Poland) with a length of 25 mm, in the amount of 4.5 kg/m^3^ of concrete mix. The Z4 blend uses CemFil Hp Macro glass fibers from the Serra-Ciments company (Barcelona, Spain) with a length of 36 mm in the amount of 1 kg/m^3^ of concrete mix. The dosage of the fibers was based on the manufacturer’s recommendations and the standard provisions [28,30]. The parameters of fibers used are summarized in Table 5 and shown in Figure 1.

### 2.2. Research Methods

The first study that was carried out was the study of the consistency of the concrete mix. The consistency of the mixture was determined by the Abrams cone fall method. The consistency test was carried out in accordance with PN-EN 12350-2:2011 Concrete mix tests—part 2: Consistency testing by the cone fall method [31].

**Figure 2:**
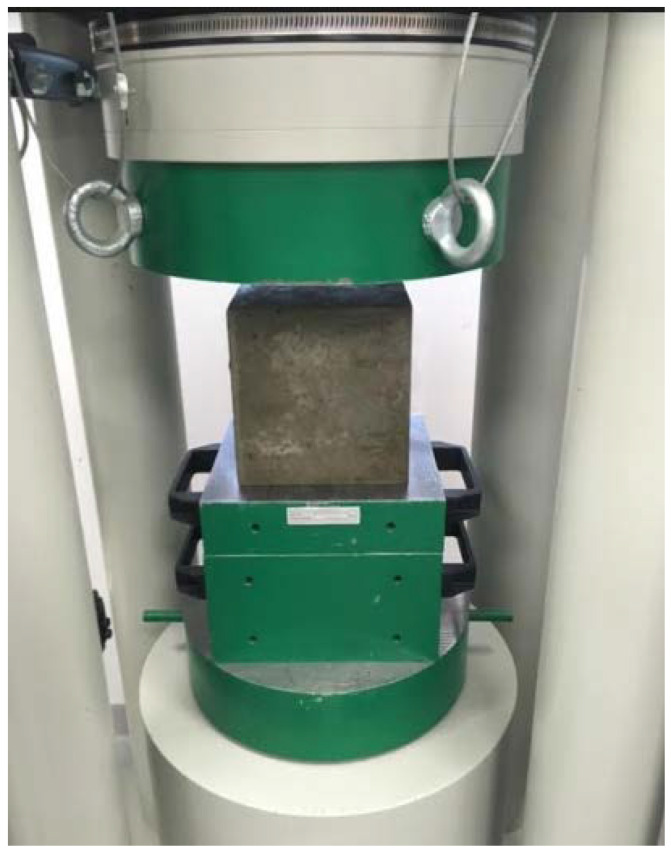
Sample during compressive strength test.

**Figure 3:**
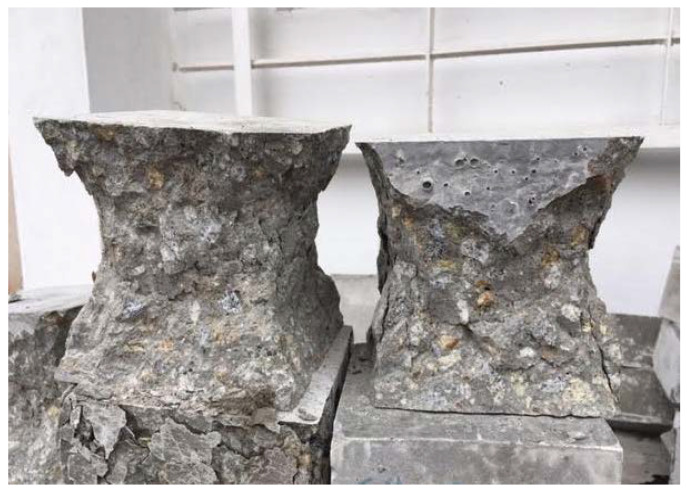
Concrete samples after compressive strength test.

Another test was the compressive strength test of cement concrete samples. This test was performed on cubic samples with dimensions of 150 mm × 150 mm × 150 mm in accordance with the provisions of PN-EN 12390-3:2019 Concrete tests—Part 3: Compressive strength of samples for testing [32]. Due to the possibility of random results, the number of samples to be tested was determined in accordance with PN-EN 206:2014 Concrete—Requirements, properties, production, and conformity [23]. The test was carried out with the use of the FORM TEST MEGA 6 3000-150 testing machine (Figure 2). Concrete samples after the compressive strength test are presented in Figure 3.

**Figure 4:**
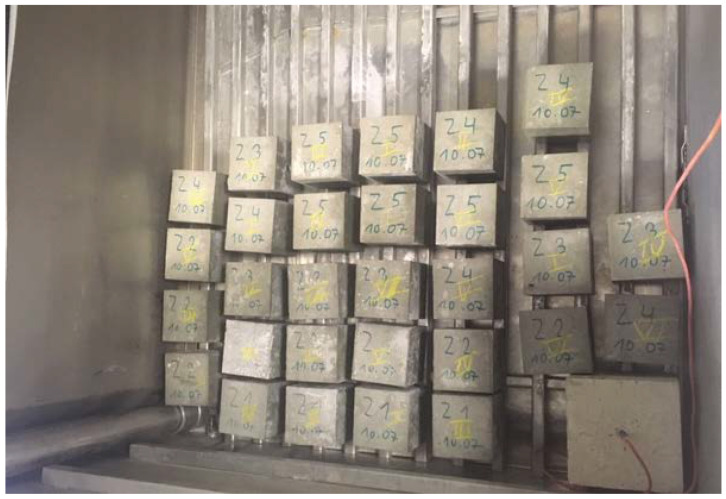
Samples prepared for frost resistance testing.

The next study aimed at determining the parameters of the designed cement concretes was the study of the frost resistance of concrete. This test was carried out by the usual method in order to verify the degree of frost resistance F assumed at the design stage of the mixture. However, this degree in the assumptions corresponds to the N index, which is equal to the number of expected years of use of the structure. The usual method makes it possible to take into account the degree of internal destruction of concrete, characterized by the decreased strength of the sample, as well as the external destruction, determined by the loss of mass of the sample. The frost resistance test was carried out in accordance with PN-88/B-06250:1988 Ordinary concrete [33]. The test was carried out with the use of the TOROPOL K-15 testing machine. Samples prepared for frost resistance testing in this device are shown in Figure 4.

**Figure 5:**
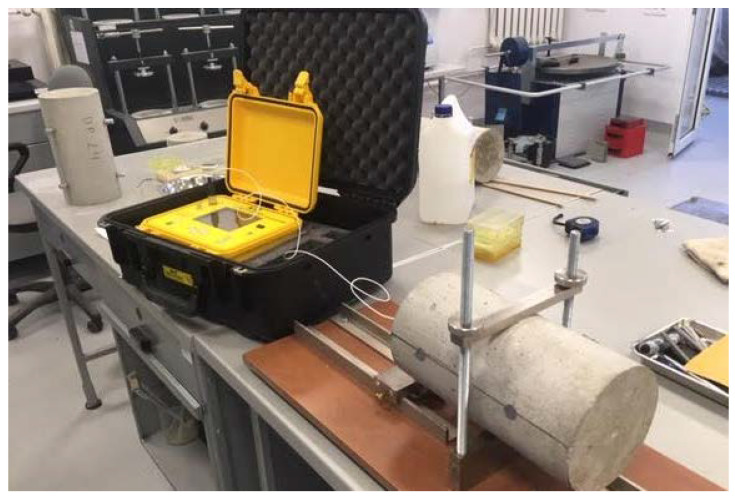
Resonance testing of the modulus of elasticity.

**Figure 6:**
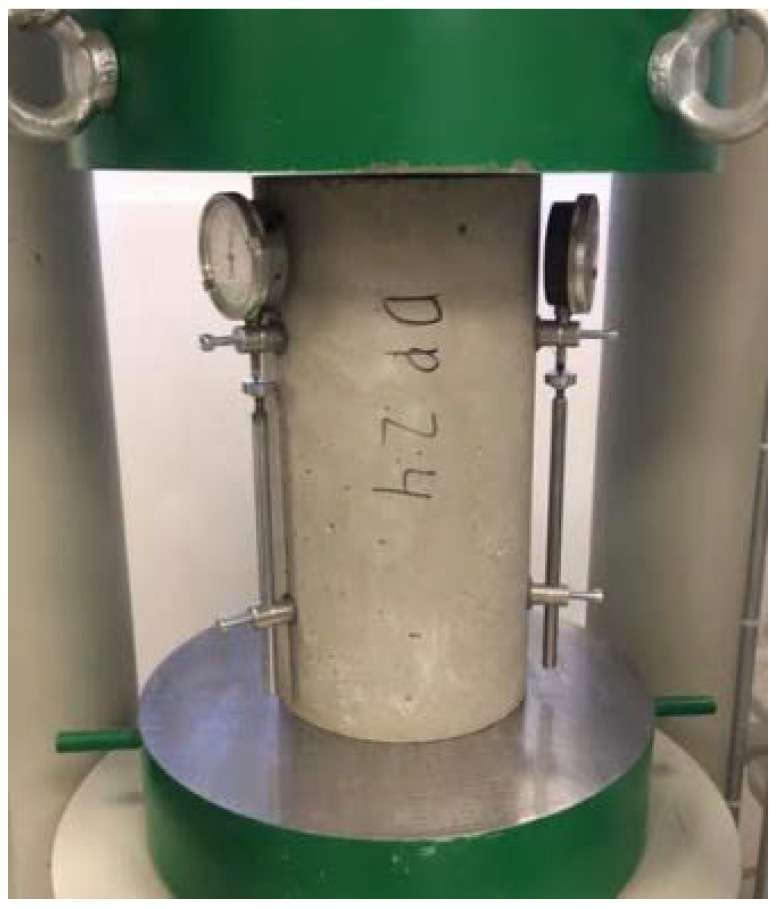
Testing of the secant modulus of elasticity at compression.

In order to determine the physical parameters of the designed cement concretes, a study of the elastic modulus was carried out. Cylindrical samples with a diameter of 150 mm and a height of 300 mm were prepared for the study. The study of elastic modulus was carried out using two methods. The first method consisted in determining the modulus of elasticity using the resonant method. In this method, the modulus of elasticity is determined by the propagation of the waves in the sample. Vibrations are caused by hitting a ball of a properly selected diameter on the base of the cylinder. Based on the measured wave, the device determines the frequency of vibrations and the modulus of elasticity of the tested material. This test is a non-destructive test of the element under study. The test was carried out with the use of the JAMES INSTRUMENT V-E-400 testing machine (Figure 5). In the second method, tests of the elastic modulus were carried out in accordance with PN-EN 12390-13:2014 Concrete tests—Part 13: Determination of the secant modulus of elasticity at compression [34]. The test was carried out with the use of the FORM TEST MEGA 6 3000-150 testing machine (Figure 6).

**Figure 7:**
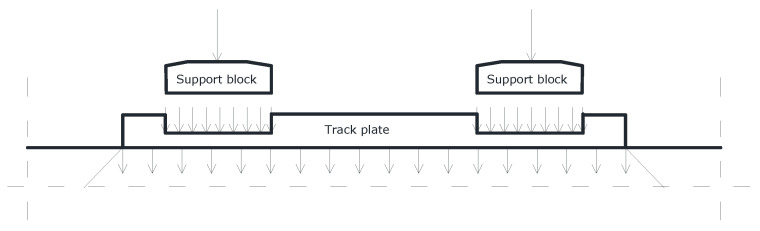
Load distribution model adopted for the test.

The main part of the experimental research was to determine the impact of the stiffness and strength of block supports on the strength of the entire ballastless surface, and thus on the strength of the track plate made of cement concrete. According to the adopted model, there are tensions of concrete in the track plate. At the same time, under the block supports, the forces from the wheeled vehicle cause the concrete to stretch downwards, and in the middle of the length of the slab the phenomenon of lifting the slab is triggered. The plate load model adopted for the cross-sectional test is shown in Figure 7.

**Figure 8:**
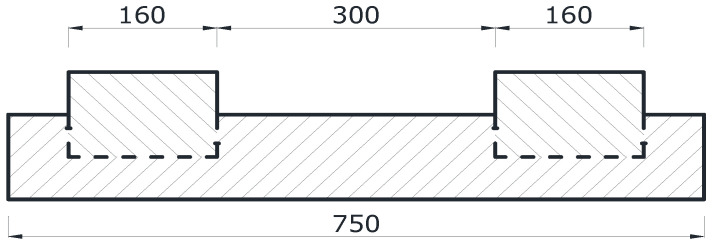
Cross-section of the laboratory sample (dimensions are described in mm).

**Figure 9:**
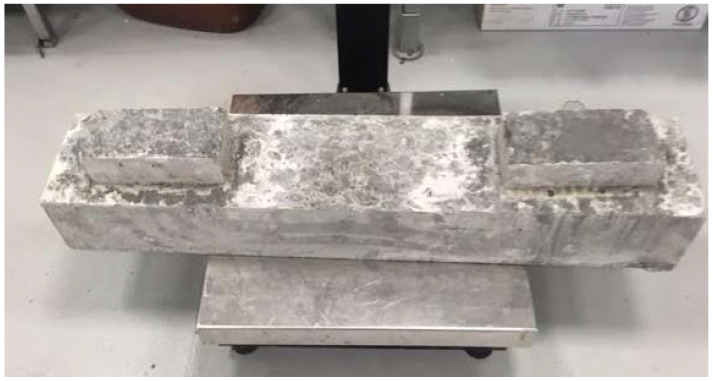
Laboratory sample prepared for testing.

Due to the research possibilities, in order to check the tensile strength when bending the track plate with embedded block supports, a laboratory sample constituting 30% of the actual dimensions was prepared. The dimensions of the sample on which the tests were carried out were 100 mm × 150 mm × 750 mm and were adapted to the forms equipped by the laboratory. Figure 8 shows a diagram of the test samples, and Figure 9 shows the sample.

**Figure 10:**
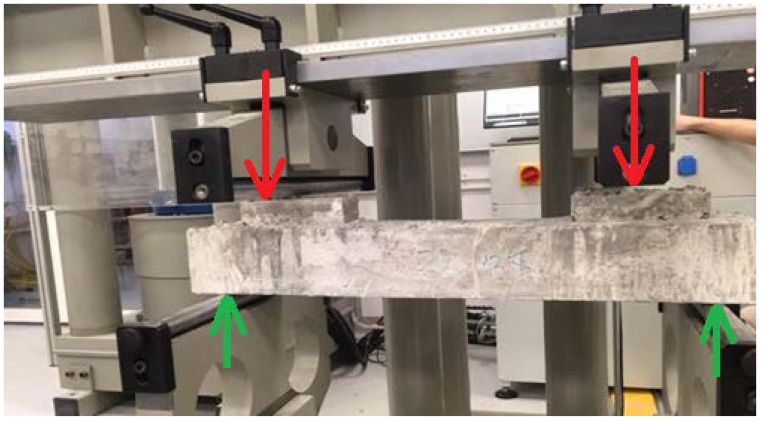
Sample in the test machine (in a model: red arrows—forces generated by the vehicle wheel, green arrows—reaction forces generated by the machine support).

The tests (Figure 10) were carried out for all cement concretes Z2–Z5 intended for use in block supports. The load was applied in the middle of the block supports so as to map the forces occurring in the structure. The support points were taken at a distance of 5 cm from the edge of the sample. The adoption of such a spacing of supports was aimed at recreating the variant of the destruction of the substructure during the operation of the railway line.

This destruction consists in settling or washing the foundation from the central part of the support. The stress value of the samples is based on the following formula.

(1) $$\sigma_{g}=\frac{P\cdot z}{2\cdot W_{g}}$$

where:

P—force acting on the sample (kN), z—distance of force from the support—0.05 m, *Wg*—index of the bending strength of the rectangular cross-section relative to the vertical axis.

**Figure 11:**
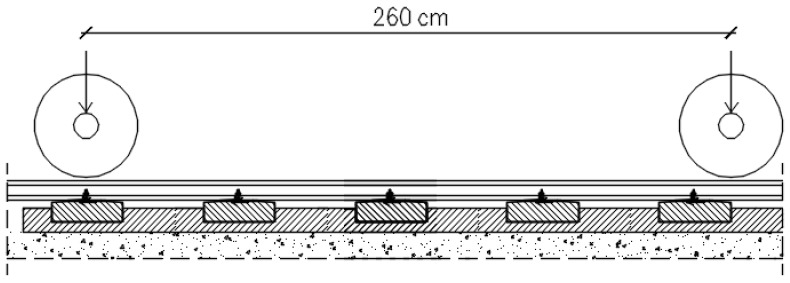
Scheme of loading the railway surface with a locomotive.

Another test aimed at determining the strength of block supports on the strength of the entire surface was to check the strength of the longitudinal section. The purpose of this test was to check the load capacity in the direction along the track, derived from the axle load of the bogie of the AEG12X locomotive, with a spacing of 2.60 m. The axle load of the locomotive is 210 kN. The plate load model adopted for longitudinal section testing is shown in Figure 11.

**Figure 12:**
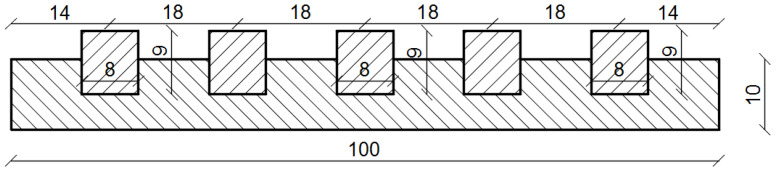
Diagram of the test sample—dimensions are expressed in cm.

**Figure 13:**
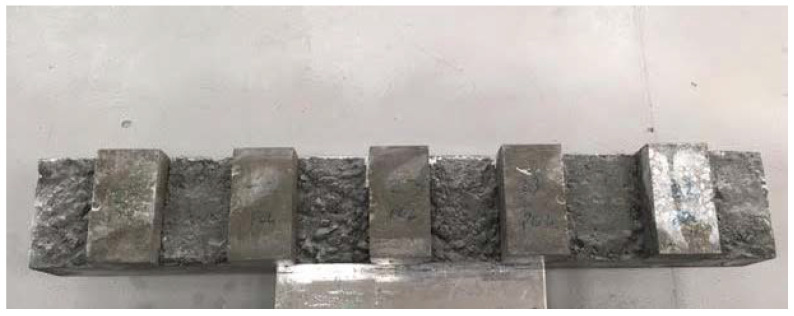
Prepared sample for bending strength testing.

In order to check the tensile strength when bending along the surface, samples measuring 10 × 15 × 100 cm were prepared. The construction of the surface was made on a scale of 0.3. It is therefore the same scale factor as in the case of cross-sectional testing. In addition to the limited availability of large molds for making samples, the choice of scale factor in this study was dictated by the capabilities of the strength testing machine. Additionally to the limited availability of large molds for making samples, the choice of scale factor in this study was dictated by the capabilities of the strength testing machine. Figure 12 shows the designed test sample with dimensions in cm. In addition, Figure 13 shows the prepared sample for testing.

**Figure 14:**
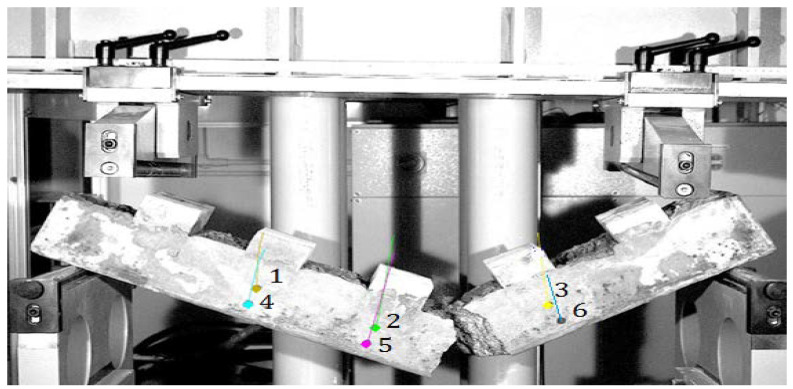
Sample after bending strength testing—dots were used to register the movement of the sample with the PHANTOM MICRO LC310 camera—not described in this article.

A sample after the bending strength testing is shown in Figure 14 (test machine is shown in Figure 10).

The research was carried out for four types of block supports. The load was applied, as in the previous test, in the middle of the block supports. Support points are adopted at a distance of 5 cm from the edge. The stress values in the samples were determined in the same way as in the previous test, i.e., according to Equation (1).

## 3. Results

### 3.1. Consistency of Concrete Mix

The results of measuring the consistency of the concrete mix are presented in Table 6.

The greatest cone fallout was observed in the case of concrete without the addition of fibers and with steel fibers. The smallest for concrete with the addition of glass fibers. The reason for this is the effect of fibers on the workability and consistency of the mixture. Fine polymer and glass fibers propagated in the mixture, improving its stability. Steel fibers, due to their greater rigidity and larger size, did not provide this property. Concrete without the addition of fibers, as a reference result, confirms this thesis.

### 3.2. Compressive Strength

**Figure 15:**
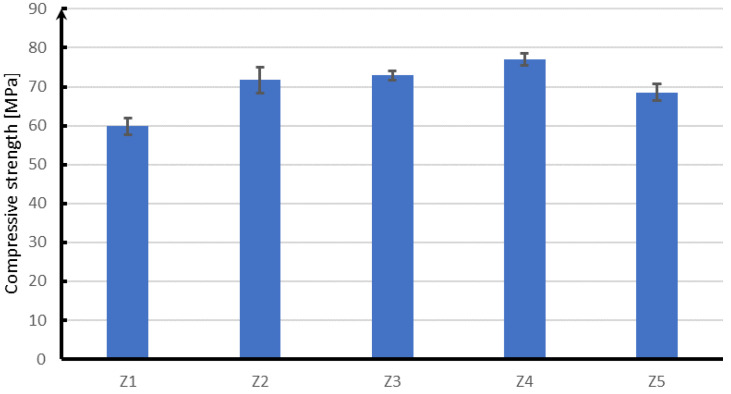
Results of the compressive strength test.

The compressive strength test was carried out for concrete used for the production of block supports and concrete used to make a concrete slab. Below in Table 7 and Figure 15 are the results of tests of concrete with additives in the form of fibers and reference concrete.

Based on the presented results, it can be concluded that the use of fibers slightly increased the compressive strength of concrete intended for block supports. The average strength of concrete without the addition of Z5 fibers is 68.5 MPa, while the strength of concrete with the addition of fibers is from 71.7 MPa for Z2 concrete, to 76.9 MPa for Z4 concrete, which is a strength increase of 4.7% to 12.3%. Although fibers have a greater impact on the increase in tensile strength than on compression, the conducted research shows that their use significantly increases the compressive strength and thus the class of concrete. In addition, it should be stated that the strength reserve is acceptable and allows us to assume that the concrete intended for the slab (Z1) and block supports (Z2–Z5) has been designed correctly.

### 3.3. Frost Resistance

The frost resistance test was carried out for concrete intended for the production of block supports and concrete intended for making a concrete slab. Below in Table 8 are the results of tests of concrete with additives in the form of fibers and reference concrete.

Based on the analysis of the obtained results, it should be concluded that all concrete samples meet the standard requirements. The permissible maximum loss in weight of samples after the frost resistance test should not exceed 5%, and the decrease in strength in relation to comparative samples not exposed to frost should not exceed 20%. All designed concretes are characterized by a loss of mass below 0.5% and a decrease in strength below 4%, which significantly exceeds the standard requirements. The research also shows that the use of fibers improved the frost resistance parameters of concrete intended for block supports in relation to the reference concrete Z5. In the case of steel fibers, the Z2 sample, the average strength decrease is reduced by 45.2% compared to the average strength decrease of concrete without fibers, and the average loss weight is reduced by 26.5%. For concrete with polymer fibers, Z3 concrete, the average strength decrease is reduced by 27.5%, and the average loss weight is reduced by 17.6%. When it comes to concrete with glass fibers, Z4 samples, the average strength decrease is reduced by 39% compared to the average strength decrease of concrete without fibers, and the average loss weight is reduced by 5.6%. Both due to the compressive strength and loss of mass after the impact of negative temperatures, concrete with the use of steel fibers looks the most advantageous. In terms of the decrease in compressive strength, Z4 concrete with glass fibers is not much worse, but in the case of a loss of mass, it is characterized by slightly more favorable results than concrete without fibers.

### 3.4. Modulus of Elasticity

As in the case of previous tests, the test of the modulus of elasticity was carried out for concrete intended for a concrete slab and concrete block supports with fibers and reference concrete without the addition of fibers. In the non-destructive method, based on the frequency of vibrations propagating in the sample, the modulus of elasticity was determined by the method of propagation of the wave in the sample. In the classical method, the modulus of elasticity was calculated on the basis of the recorded deformation of the sample during loading according to Formula (2). The results of the elastic modulus for individual samples determined by method one are summarized in Table 9 and by the classical method in Table 10.

(2) $$E=\frac{\Delta \sigma }{\Delta \varepsilon }\;$$

where:

∆*σ*—compressive stress increment (MPa);

∆*ε*—sample deformation.

The results obtained by the resonant method are higher, but the difference between the results in relation to the classical method is about 2% to 4%. In both test methods used, the influence of the addition of fibers on the modulus of elasticity of cement concrete can be seen. The highest modulus of elasticity in both resonance and load tests is characterized by samples with glass fibers, whose modulus of elasticity is greater than concrete without fibers in the resonant method by 4.8% and in the classical method by 3.9%. Slightly worse are samples with polypropylene fibers, whose elastic modulus is higher by 4.2% in the resonance method and in the classical method by 3.8%. Steel fibers, whose elastic modulus is higher by 1.4% in the resonance method, and by 1.2% in the classical method, have the least positive effect.

### 3.5. Tensile Strength of the Cross-Section of the Ballastless Railway Surface

In order to determine the influence of the strength parameters of block supports on the concrete slab, tensile strength tests at bending described in Section 2 of the laboratory model reflecting the actual track layout in the cross-section, with a scale factor of 0.3, were carried out. Z1 concrete was used as the material of the concrete slab and for block supports both concrete without Z5 fibers and with the addition of Z2 steel fibers, Z3 polypropylene and Z4 glass fibers. The results of the tensile strength at bending are shown in Table 11.

Based on the analysis of the presented results, it should be concluded that the fibers used in the concrete of block supports affect the tensile strength when bending the concrete slab. The highest tensile strength during bending has a plate with supports in which glass fibers are used, and the smallest with supports in which concrete without the addition of fibers is used. Glass fibers in concrete intended for block supports increased the tensile strength of the plate at bending by 17.5% compared to block supports in which no fibers were used. Steel fibers increased the tensile strength of the plate at bending by 10.7%, and polypropylene fibers by only 1.2%. It should also be noted that the degree to which individual fibers affect does not depend either on the compressive strength of the cement concrete or on the modulus of elasticity. The key in this case is how the material of block supports cooperates with the concrete slab in the transfer of loads.

### 3.6. Tensile Strength of the Longitudinal Section of the Ballastless Railway Surface

Another important study is to determine the influence of the material from which block supports were made on the tensile strength of the concrete slab in a longitudinal system. In this case, as before, the tests were performed on laboratory samples prepared on a scale of 0.3. The research was carried out for a concrete slab using block supports without fibers (Z5) and with the addition of fibers (Z2–Z4). The results of the study are presented in Table 12.

In the case of the longitudinal system, one can also notice increased tensile strength when bending the concrete slab after using reinforcing fibers in block supports. However, in the longitudinal arrangement, the difference in the results is not as large as in the case of the transverse arrangement. In the longitudinal system, block supports with glass fibers also had the best effect on increasing the tensile strength when bending the concrete slab and increased it by 8.6%. For the transverse system, it was 17.5%. Polypropylene and steel fibers increased the strength of the concrete slab by 7.5% and 7.1%, respectively. In the case of the longitudinal system, you can see a much more favorable effect of polypropylene fibers on the tensile strength when bending the concrete slab than in the case of the transverse system, where this benefit was only 1.2%. In the case of the longitudinal system, you can see a slightly more correlation of the impact of the increase in tensile strength when bending the concrete slab with the compressive strength of the concrete block supports. However, it is not possible to talk about a full correlation due to the value of standard deviations of the obtained results, which indicate that each type of fibers used equally affects the tensile strength of the concrete slab.

## 4. Discussion

**Figure 16:**
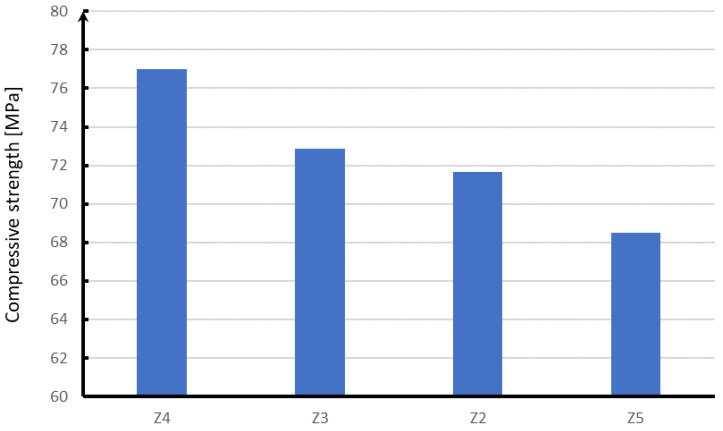
Results of the compressive strength test.

**Figure 17:**
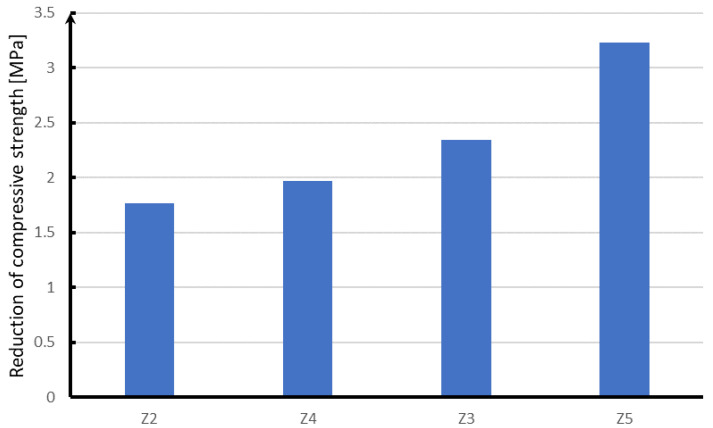
Results of the frost resistance test.

**Figure 18:**
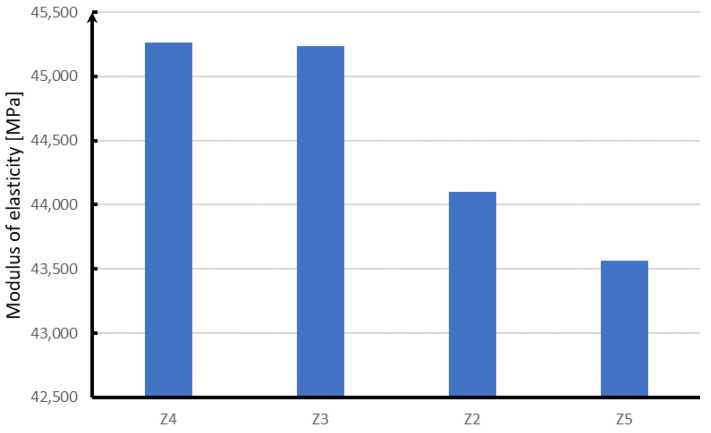
Results of the elastic modulus test.

**Figure 19:**
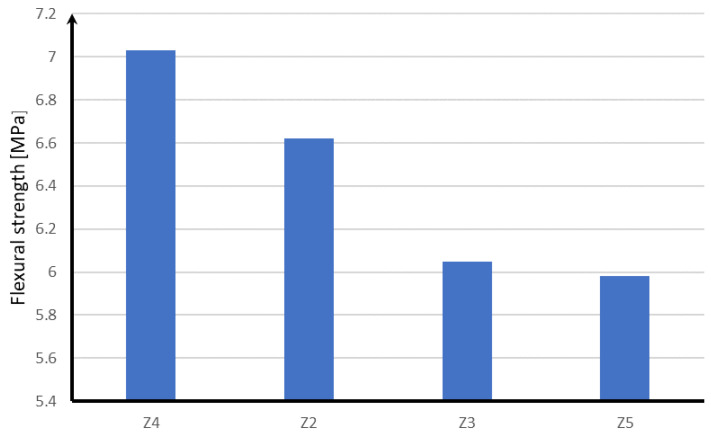
Stresses in the track plate depending on the fibers used in the block supports.

On the basis of the conducted research, it can be concluded that individual strength results depend on the reinforcing fibers used. Samples with the addition of glass fibers are the most advantageous in the strength tests carried out, which are shown in Figure 16, Figure 17, Figure 18 and Figure 19.

In the case of compression (Figure 16), the next in terms of strength are samples with the addition of polypropylene fibers, which in relation to glass fibers are characterized by a strength lower by about 4 MPa. On the other hand, steel fibers have a strength lower by about 5 MPa than glass fibers. The lowest compressive strength of about 68 MPa is characterized by concrete without reinforcing fibers.

In the case of frost resistance of cement concrete (Figure 17), samples with the addition of steel fibers, whose strength drop is 1.8 MPa, are the most advantageous. Glass fibers with the most favorable compressive strength are also characterized by the highest strength of samples subjected to frost, but their strength drop is greater than that of steel fibers and amounts to almost 2 MPa. Next are samples with the addition of polypropylene fibers, which are characterized by a decrease in strength equal to 2.3 MPa. The least favorable are samples without the addition of fibers, whose strength drop is over 3.2 MPa.

Analyzing the modulus of elasticity of concrete samples (Figure 18), it can be concluded that, as in the case of compressive strength, the highest module is characterized by samples with the addition of glass and polypropylene fibers. The difference between the obtained results for both fibers is within the limit of statistical error. Steel fibers are characterized by a modulus of elasticity lower by about 1000 MPa, while samples without the addition of fibers are characterized by a module lower by about 2000 MPa.

Taking into account the influence of reinforcing fibers on the work of the concrete slab supporting the block supports, it should be noted that the most advantageous variant is the use of glass fibers in block supports, thanks to which the concrete slab is characterized by the highest tensile strength in both longitudinal and transverse systems. In the case of steel fibers, the tensile strength of the concrete slab at bending is reduced by about 0.4 MPa. For polypropylene fibers, the strength is lower by about 1 MPa. The least favorable effect on the strength of the concrete slab is the use of block supports without the addition of fibers, which is shown in Figure 19.

The analyses carried out above show that the most advantageous in terms of strength and proper work of the surface is the use of glass fibers in block supports, which at the same time increase the strength of concrete intended for block supports and increase tensile strength when bending the concrete slab constituting support for block supports.

Having in mind the above discussion one may conclude what follows.

Table 1, Table 2, Table 3 and Table 4 present the properties and compositions of concrete mixtures accepted for the production of samples without the addition of fibers (Z1 and Z5) and with the addition of fibers (Z2–Z4). All samples (Z1–Z5) were subjected to strength tests. Some of the tests concerned the samples themselves. The concrete mixes (Z1–Z5) were then used for elements of the railway surface, made on a laboratory scale. After that, additional tests were made for properly made aggregated railway track elements. The values of the tested physical quantities differed, and, moreover, their distribution was not the same in each individual test. The sequences of positions of individual material samples or elements made of them are different in different Table 1, Table 2, Table 3 and Table 4 and different diagrams (Figure 16, Figure 17, Figure 18 and Figure 19). Hence, the resulting material with various additives has different properties. This applies both to the compressive strength of the material samples themselves and to the bending strength of the fixed elements of the railway track, which are made of various materials with additives. The study of the influence of low temperatures on the change in the technical parameters of individual materials and elements made of them, as it was presented, is also very important. This factor in engineering practice must be taken into account, especially in climatic conditions, in which specific structures will be exposed to sub-zero temperatures or to cyclical temperature changes.

Finally, it should be stated that when changing the rail support in the transition railway sections, in order to eliminate the threshold effect, it is necessary to use a proper sequence of various reinforcing fibers in the set of railway concrete elements, which will properly affect the total rigidity of the track system in the transition zone.

## 5. Conclusions

This article is a continuation of the research described in [13], in which the phenomena occurring in the zones of changes in the technology of the railway surface were analyzed. In the transition zones from the ballast surface to the ballastless surface of the railway line, there are jumping changes in the strength parameters of the materials from which the elements of the surface are made. This adversely affects the dynamic interactions of the track–rail vehicle system. This phenomenon, called the threshold effect, was considered in [13], where reinforcements of materials on the ballast surface side were proposed, partially reducing the adverse effects of the threshold phenomenon. This paper presents how it is possible to influence the gradual change of strength parameters of materials on the ballastless side using concrete with various, gradually changing additives for the construction of pavement elements. As a consequence, the strength parameters in the transition zone, from ballast to ballastless surface at the entrance to the engineering facility and from ballastless to ballast surface when leaving the zone of the engineering facility, can be constructed in such a way that the change in the strength parameters of the materials used is not incremental, but rather changes continuously. It has been shown that this would be possible by using various concrete additives so that in a zone of appropriate length, the strength properties of the pavement elements change as continuously as possible.

The next step in the test cycle could be the use of an experimentally proven computational model [13], which would exploit the possibilities of affecting the ballast part of the surface in the transition zone [13] and the ballast-free part, discussed in this article, so as to propose design solutions in which the dynamic vehicle-track interactions will be limited as much as possible.

## Figures and Tables

**Table 1:** Properties of cements.

Parameter	Unit	CEM I 42.5 N/NA WARTA	CEM I 42.5 R CEMEX
Compressive strength			
after 2 days	MPa	17.9	25.6
after 28 days	MPa	50.5	54.3
Bending strength			
after 2 days	MPa	3.7	-
after 28 days	MPa	8.3	-
Setting time:			
beginning	min	200	187
the end	min	280	248
Specific surface	cm^2^/g	3207	3746
Cement shrinkage	mm/m	0.46	-
Loss of roasting	%	3.80	3.24
Chemical composition:			
CaO	%	63.69	-
MgO	%	1.07	-
SiO_2_	%	20.19	-
Al_2_O_3_	%	4.49	-
Fe_2_O_3_	%	2.78	-
SO_3_	%	2.36	2.98
Na_2_Oeq	%	0.54	0.60
Cl	%	0.032	0.079

**Table 2:** Assumptions for concrete mixes.

Parameter	Concrete for Track Slab	Concrete for Block Supports
Compressive strength class	C35/45	C50/60
Exposure class to carbonation corrosion	XC4	XC4
Frost aggression exposure class	XF1	XF1
Degree of frost resistance	F150	F125
Required cement grade	CEM 42.5	CEM 42.5
Maximum cement content [kg]	400	450
Required consistency	S3	S2
Maximum absorbability of concrete	5%	4%
Water-cement indicator w/c	<0.5	<0.45

**Table 3:** Concrete mix design for track slab (Z1).

Ingredient	Content [kg]
Cement CEM I 42.5 N/NA from WARTA company	390
Fine aggregate Sand 0/2 from Żabiny company	517
Coarse aggregate 1 Granite 2/8 from Graniczna company	497
Coarse aggregate 2 Granit 8/16 from Graniczna company	919
Water	148
Plasticizer Version of Viscocrete	3.1
Aerator Sica AerPro 3	0.6

**Table 4:** Design of concrete mix for block supports.

Ingredient	Content [kg]
Cement CEM I 42.5 R from CEMEX company	420
Fine aggregate Sand 0/2 from Żabiny company	486
Coarse aggregate 1 Granite 2/8 from Graniczna company	523
Coarse aggregate 2 Granit 8/16 from Graniczna company	860
Water	155
Plasticizer Version of Viscocrete	3.3
Aerator Sica AerPro 3	0.7

**Table 5:** Parameters of fibers.

Parameter	Steel Fibers	Polymer Fibers	Glass Fibers
Diameter	1.0 mm ± 10%	0.6 mm	0.01 mm
Length	50 mm ± 10%	25 mm ± 10%	36 mm ± 10%
Dosage	25–35 kg/m^3^	3–9 kg/m^3^	0.5–2 kg/m^3^
Tensile strength	1100 MPa	550–650 MPa	2500 MPa

**Table 6:** Results of the measurement of cone fall.

Concrete Type	Cone Fall [mm]	Consistency Class
Z1	140	S3
Z2	80	S2
Z3	70	S2
Z4	60	S2
Z5	80	S2

**Table 7:** Results of the compressive strength test.

Concrete Type	Compressive Strength [MPa]	Standard Deviation [MPa]
Z1	59.80	2.12
Z2	71.67	3.26
Z3	72.87	1.14
Z4	76.98	1.52
Z5	68.50	2.14

**Table 8:** Results of the frost resistance test.

Concrete Type	Average Weight Loss (%)	Compressive Strength of the Comparative Samples (MPa)	Compressive Strength of Samples after Frost Resistance Test (MPa)	Average Strength Decrease (%)
Z1	0.21	59.8	58.2	2.67
Z2	0.25	73.3	72.0	1.77
Z3	0.28	73.1	71.4	2.34
Z4	0.32	76.3	74.9	1.97
Z5	0.34	71.3	69.0	3.23

**Table 9:** Results of the resonance test of the modulus of elasticity.

Concrete Type	Medium Modulus of Elasticity (MPa)	Standard Deviation (MPa)
Z1	40,467	550.8
Z2	45,433	115.5
Z3	46,667	288.7
Z4	46,933	208.2
Z5	44,800	300.0

**Table 10:** Results of the classical elastic modulus test.

Concrete Type	Medium Modulus of Elasticity (MPa)	Standard Deviation (MPa)
Z1	38,567	472.6
Z2	44,100	264.6
Z3	45,233	416.3
Z4	45,267	321.5
Z5	43,567	321.5

**Table 11:** Results of the tensile strength test at bending of the Z1 concrete beam depending on the reinforcing fibers used in the block supports.

Concrete Type	Average Tensile Strength (MPa)	Standard Deviation (MPa)
Z2	6.62	0.42
Z3	6.05	0.10
Z4	7.03	0.34
Z5	5.98	0.16

**Table 12:** Results of the tensile strength test at bending of the Z1 concrete beam depending on the reinforcing fibers used in the block supports.

Concrete Type	Average Tensil Strength (MPa)	Standard Deviation (MPa)
Z2	7.82	0.36
Z3	7.85	0.27
Z4	7.93	0.39
Z5	7.30	0.14

## Data Availability

The data presented in this study are available upon request from the corresponding author.
